# Postoperative Complications After Cytoreductive Surgery for Advanced Ovarian Carcinoma: A Single-Center Analysis Exploring the Value of the Comprehensive Complication Index and the Predictors of High Complications Burden

**DOI:** 10.1245/s10434-025-17619-9

**Published:** 2025-06-24

**Authors:** Tommaso Bianchi, Tommaso Grassi, Elena De Ponti, Giorgia Pecis Cavagna, Martina Bombelli, Daniele Lugotti, Filippo Testa, Mauro Totis, Gaetano Trezzi, Fabio Landoni, Robert Fruscio

**Affiliations:** 1 Department of Medicine and Surgery, University of Milano-Bicocca, Milano, Italy; 2 UO Gynecology, Fondazione IRCCS San Gerardo dei Tintori, Monza, Italy; 3 Medical Physics, Fondazione IRCCS San Gerardo dei Tintori, Monza, Italy; 4 UO Surgery, Fondazione IRCCS San Gerardo dei Tintori, Monza, Italy

**Keywords:** Ovarian carcinoma, CCI, Complications, Cytoreductive surgery, Clavien–Dindo

## Abstract

**Background:**

The comprehensive complication index (CCI) reflects the overall patient complication burden on a 0–100 scale. This single-institution retrospective analysis explores the accuracy of CCI in describing complications following cytoreductive surgery for advanced high-grade ovarian carcinoma (HGOC) and aims to identify predictive factors of high complication burden.

**Patients and Methods:**

In total, 304 patients who underwent cytoreductive surgery for FIGO stage IIIA–IVB HGOC at our institution from 2015 to 2023 were analyzed. Each complication’s severity was graded using the Clavien–Dindo classification. The CCI was used to quantify the global complications burden, and patients were stratified into three groups: CCI-low (< 26.2), CCI-intermediate (26 ≤ CCI < 33.7), and CCI-high (≥ 33.7).

**Results:**

Of the 200 patients (65.8%) with at least one complication, 127 (41.8%) were CCI-low, 32 (10.5%) CCI-intermediate, and 41 (13.5%) CCI-high. Median hospitalization duration (*p* < 0.0001) and readmission rates (*p* < 0.0001) correlated with CCI categories, reflecting increased CCI scores with greater surgical complexity, as assessed by the Aletti surgical complexity score (SCS). Univariate analysis showed a significant association between CCI-high and FIGO stage, surgical complexity, diaphragmatic procedures, multiple bowel resections, length of surgery and intraoperative blood loss. Multivariate analysis confirmed FIGO stage (odds ratio [OR] 2.57), multiple bowel resections (OR 5.61), and blood loss (OR 1.93) as independent risk factors for high complication burden.

**Conclusions:**

The CCI is a good descriptor of postoperative complications in patients undergoing cytoreductive surgery for advanced HGOC by integrating both the severity and number of complications into a single, easily usable, and intuitive quantitative score. FIGO stage, multiple bowel resections, and blood loss—but not surgical timing—are independent predictors of a high complication burden.

The absence of residual disease (RD) after cytoreductive surgery is one of the most important prognostic factors in the treatment of advanced high-grade ovarian carcinoma (HGOC).^1–3^ Primary debulking surgery (PDS) represents the standard treatment in these patients. In patients of older age, not fit enough to undergo extensive surgery and/or with non-optimally resectable tumors, neoadjuvant chemotherapy (NACT) followed by interval debulking surgery (IDS) is an oncologically safe alternative to PDS.^4–7^

The aim of removing all macroscopically visible tumor has shifted the surgical paradigm from the pelvis-oriented surgery of the 20th century to extended abdominal cytoreductive procedures, including systematic peritonectomy, diaphragmatic stripping and/or resections, multiple bowel resections, and splenic–pancreatic and liver procedures.^1,8–13^

The impact of extensive cytoreductive surgery on postoperative morbidity and, ultimately, the relationship between major postoperative complications and oncologic outcomes are a matter of debate. In addition, the way postoperative complications are reported varies among studies, impairing the possibility of accurately evaluating predictive factors of major complications and their actual impact on morbidity and oncologic outcomes.^13–18^

In contrast to other complication grading systems, which usually consider the most severe complication that occurred in each patient, the comprehensive complication index (CCI)^19^ considers each complication, reflecting the overall patient complication burden, using a 0–100 scale. A recently published paper by Kengsakul et al.^20^ validated the CCI in the prediction of postoperative morbidity outcomes in 300 patients receiving cytoreductive surgery for FIGO stage IIIB–IVB ovarian carcinoma. However, most patients (85%) in that series underwent IDS, leaving the door open for further research in patients undergoing PDS.

Since surgical morbidity and postoperative recovery are determined not only by the occurrence of single life-threatening complications but also by the sum of multiple minor events and that PDS is the treatment of choice in patients with advanced HGOC, we performed this single-center retrospective analysis to explore the reliability of the CCI in the description of complications following cytoreductive surgery for advanced HGOC and to identify predictive factors of postoperative complications.

## Patients and Methods

### Study Design

This single-institution retrospective study explores the rate of postoperative complications, the reliability of the CCI as a descriptor of surgical complications, and the predictors of severe complication burden in patients treated for advanced HGOC in an ESGO-accredited tertiary gynecologic oncology center.

The study received approval from the institutional review board (IRB) of our institution, and patients’ informed consent was obtained.

### Study Population, Treatment Choice, and Data Collection

All patients with newly diagnosed FIGO stage IIIA–IVB, non-mucinous HGOC who received either PDS or IDS at our institution from 1 January 2015 to 31 December 2023 were included in this analysis, irrespective of the RD after surgery. Only patients who were operated on with cytoreductive intent for advanced-stage HGOC were included; patients receiving only diagnostic surgery or staging surgery for early-stage HGOC were excluded.

All patients scheduled for either PDS or IDS were managed perioperatively according to the ERAS guidelines.^21^ All surgical procedures were carried out or supervised by gynecologic oncology surgeons with specific expertise in the treatment of advanced HGOC. The primary treatment was determined by the multidisciplinary tumor board of our institution according to disease resectability and the patient’s eligibility for a major surgical procedure. Patients considered resectable at preoperative imaging, and with permissive performance status (ECOG 0–2), were candidates for PDS. For those patients not fit enough to receive extensive surgical procedures and/or with a non-optimally resectable tumor, a course of platinum-based NACT was indicated as primary treatment followed by IDS.

Patients’ demographic, surgical, clinical, and pathological data were collected from internal electronic records. The tumor’s related data included histology, tumor stage according to the FIGO 2014 classification,^22^ and the tumor’s intra-abdominal extension was assessed with the peritoneal cancer index (PCI).^23^ The surgical timing, either PDS or IDS, the RD after cytoreductive surgery, the surgical complexity, the type of surgical procedures, the length of surgery, and intraoperative blood loss were the recorded treatment-related variables. RD was defined as follows: (i) R0 if complete resection was achieved; (ii) R1 in the case of RD < 1cm; and (iii) R2 in the case of RD > 1cm. Surgical complexity was defined according to the Aletti surgical complexity score (SCS).^24^

### Surgical Complications

The rate of surgical complications, re-interventions, and hospital readmissions occurring within 30 days after surgery were recorded. The length of hospital stay and the median time to the first adjuvant chemotherapy cycle were also documented.

Surgical complications were defined as any deviation from a regular postoperative course requiring only immediate postoperative fluid integration and analgesics. Even those events necessitating minor medical intervention (i.e., iron supplementation for mild–moderate anemia) were recorded as complications. The severity of each complication was classified according to the grading reported in the Clavien–Dindo (CD) classification.^25^ Complications were classified as surgical site, pulmonary, gastrointestinal, urinary, and hemorrhagic. No cardiac complications were observed and were therefore excluded from the above-mentioned categories. Hemorrhagic complications were sub-classified depending on the subsequent anemia severity; anemia was considered moderate if only iron supplementation was required (CD grade 1), whereas it was classified as severe if blood transfusion(s) was needed (CD grade 2).

For each patient, the burden of surgical complications was calculated using the open access CCI online calculator (www.cci-calculator.com) and subsequently stratified as follows. The high complication burden group (CCI-high) was designed to include all patients with a CCI ≥ 33.7, comprising those patients with at least a complication of grade IIIb or greater, or a combination of grade I–IIIa complications leading to a CCI ≥ 33.7. The cutoff for this subgroup was chosen to include all patients who needed at least a re-intervention or experienced multiple minor (grade I–IIIa) complications with a total burden as heavy as at least a re-intervention. A low complication burden group (CCI-low) was identified in patients with a CCI < 26.2.

The cutoff for this subgroup was chosen to exclude any patient who experienced at least a surgical, endoscopic, or radiological procedure not requiring general anesthesia (grade IIIa). Using this cutoff, patients who experienced up to nine grade I complications (CCI = 26), fewer than two grade II complications (CCI = 29.2), or a combination of grade II and grade I complications resulting in a CCI < 26.2 were included. All patients with a CCI ≥ 26.2 and < 33.7 were included in the intermediate complication burden group (CCI-intermediate).

### Statistical Analysis

Discrete variables were expressed in fractions and compared using Fisher’s exact or Chi-squared tests. Continuous variables were expressed as medians and compared using the sum rank test. Cox regression models were used to evaluate the impact of different covariates on the incidence of complications. All statistical tests were two-sided. *Stata software 9.0* was used to perform all statistical analyses and a level of *p* < 0.05 was adopted for significance.

## Results

### Study Population: Baseline Characteristics and Treatment Related Procedures and Surgical Complications

In total, 304 patients with newly diagnosed FIGO stage IIIA–IVB, non-mucinous HGOC referred to our institution for primary surgical treatment during 2015–2023 were included in the study.

Table 1 reports patients’ baseline characteristics. The median age at diagnosis was 65 years and most patients had a good general health status, with favorable ASA score (ASA 1–2) and good performance status (ECOG 0–1) in 67.1% and 97.4% of patients, respectively. Only 27% of patients had two or more pre-existing comorbidities and 53.3% of patients had at least one previous abdominal surgical procedure. Median preoperative hemoglobin (Hb), total serum proteins, and preoperative serum albumin were 12.2 g/dL, 7 g/dL, and 3.85 g/dL, respectively. In 17.4% and 24.7% of patients, preoperative total serum proteins and preoperative serum albumin levels were below the range of normality, respectively.

**Table 1:** Clinical and demographic baseline characteristics of patients

Variable		*N*	%
Age	Median (IQR)	65	57–72
BMI (kg/m^2^)	Median (IQR)	24	21–27
ASA	ASA1	5	1.6%
	ASA2	199	65.5%
	ASA3	98	32.2%
	ASA4	2	0.7%
ECOG	0	204	67.1%
	1	92	30.3%
	2	8	2.6%
Comorbidities	Other previous malignancies	34	11.2%
	Two or more comorbidities	82	27.0%
Previous surgery	No	142	46.7%
	Yes	162	53.3%
Preoperative Hb (g/dL)	Median (IQR)	12.2	11.0–13.1
Preoperative PLT (10^3^/uL)	Median (IQR)	286	216–373
Preoperative total serum proteins (g/dL)	Median (IQR)	7.0	6.6–7.4
	< 6.4	53	17.4%
Preoperative serum albumin (g/dL)	Median (IQR)	3.85	3.36–4.15
	< 3.5	75	24.7%
Histotype	High grade serous	267	87.8%
	High grade endometrioid	10	3.3%
	Clear cell	6	2.0%
	Carcinosarcoma	15	4.9%
	Other	6	2,0%
FIGO stage	IIIA	17	5.6%
	IIIB	21	6.9%
	IIIC	169	55.6%
	IVA	19	6.3%
	IVB	78	25.7%
PCI	Median (IQR)	11	7–16
Surgical treatment	PDS	199	65.5%
	IDS	105	34.5%
Residual disease	R0	193	63.5%
	R1	77	25.3%
	R2	34	11.2%
Surgical complexity score	Median (IQR)	4	3–7
	SCS 1–3 (low)	113	37.2%
	SCS 4–7 (intermediate)	121	39.8%
	SCS > 7 (high)	70	23.0%
Surgical procedures	Diaphragmatic procedures	88	28.9%
	Splenectomy	22	7.2%
	Liver procedures	18	5.9%
	Lymphadenectomy	64	21.1%
	Bowel resection	105	34.5%
	Multiple bowel resections	33	10.9%
Blood loss (mL)	Median (IQR)	500	300–800
Length of surgery (min)	Median (IQR)	225	176–294
Length of stay (days)	Median (IQR)	6	4–9

Most patients had FIGO stage IIIC and IVB disease (55.6% and 25.7%), with a median PCI of 11. Among patients with FIGO stage IVB, extra-abdominal disease was diagnosed in 21 (26.9%) patients.

Overall, 199 (65.5%) patients received PDS, and 105 received IDS (34.5%). R0 was achieved in 63.5% of patients, whereas 25.3% and 11.2% had R1 and R2 at the end of surgery, respectively. Comprehensively, 88,8% of patients received optimal (RD < 1cm) cytoreduction.

The median SCS was 4 (IQR 3–7). Intermediate and high complexity surgery was performed in 39.8% and 23% of patients, whereas only a minority received a low complexity procedure (37.2%). Diaphragmatic procedures, splenectomy, liver procedures, and pelvic and/or para-aortic lymphadenectomy were performed in 28.9%, 0.2%, 5.9%, and 21.1% of patients, respectively. Among the 105 patients (34.5%) undergoing a bowel resection, 33 (31.4%) received more than one resection. The median duration of surgery was 225 min (IQR: 176–294 min) and the median blood loss was 500 mL (IQR: 300–800 mL).

The overall rate of postoperative complications was 65.8%, with 200 patients experiencing at least one complication (Table 2). Postoperative re-intervention within 30 days after cytoreductive surgery occurred in 23 (7.6%) patients, whereas 28 (9.2%) patients experienced postoperative hospital readmission.

**Table 2:** Postoperative complications

		*N*	%
Postoperative complications < 30 days	Yes	200	65.8%
	No	104	34.2%
Postoperative re-intervention < 30 days	Yes	23	7.6%
	No	281	92.4%
Postoperative hospital re-admission	Yes	28	9.2%
	No	276	90.8%
Peri- and postoperative blood transfusions	Yes	121	39.8%
	No	183	60.2%
Patients with at least one peri- and postoperative complication < 30 days	Grade I	72	23.7%
	Grade II	149	49.0%
	Grade III	41	13.5%
	Grade IV	4	1.3%
	Grade V	3	1.0%
CCI	Median (IQR)	12.2	0–22.9
	Patients with CCI < 26.2	127	41.8%
	Patients with CCI ≥ 26.2 < 33.7	32	10.5%
	Patients with CCI ≥ 33.7	41	13.5%
Surgical site complications	Infection	13	4.3%
	Diastasis (vaginal)	2	0.7%
	Diastasis (abdominal)	6	2.0%
Pulmonary	Pulmonary TVE	8	2.6%
	Pneumonia	4	1.3%
	Pleural effusion	20	6.6%
Anemia	Moderate	41	13.5%
	Severe	114	37.5%
Intestinal complications	Sub-occlusion	9	3.0%
	Occlusion	4	1.3%
	Anastomotic leak	8	2.6%
Urinary complications	UTI	7	2.3%
	Ureteronephrosis	1	0.3%
	Ureteral lesion/fistula	2	0.7%
	AKI	2	0.7%

Minor complications (grade I–II) were the most frequently recorded, with 23.7% and 49% of patients experiencing at least one grade I and one grade II complication, respectively. Major surgical complications occurred in a minority of patients, with 41 patients (13.5%) developing at least one grade III complication and 4 patients necessitating intensive care unit (ICU) support (grade IV). In total, 3 patients (1%) died of postoperative complications.

Regarding the type of complications, 155 patients (51%) experienced anemia due to intraoperative blood loss and/or postoperative hemorrhagic complications, 21 patients (7%) had a surgical site complication, 32 (10.5%) a pulmonary complication, 12 (4%) and 21 (7%) a urinary and gastrointestinal (GI) event, respectively.

Among the 155 patients with a hemorrhagic complication, severe anemia requiring blood transfusion(s) occurred in 114 patients (37.5%). Among the 21 patients experiencing a GI event, an anastomotic leak occurred in 8 cases out of 105 undergoing at least one bowel resection, with a global anastomotic dehiscence incidence of 7.6%. Pleural effusion was the most frequent pulmonary complication and was recorded in 20 patients (6.6%), which required percutaneous drainage in 9 cases (2.9%). Regarding surgical site complications, abdominal wall wound infection was recorded in 13 patients (4,3%), whereas vaginal and abdominal suture diastasis occurred in 2 (0.7%) and 6 (2%) patients, respectively. Among urinary complications, urinary tract infections were the most frequently recorded (2.3%).

### Surgical Morbidity, Surgical Complexity, and Complication Burden

Overall, the median CCI was 12.2 (IQR 0–22.9). A CCI-low burden was observed in 127 patients (41.8%), whereas a CCI-intermediate and CCI-high burden were observed in only 32 (10.5%) and 41 (13.5%) of patients, respectively.

**Fig. 1:**
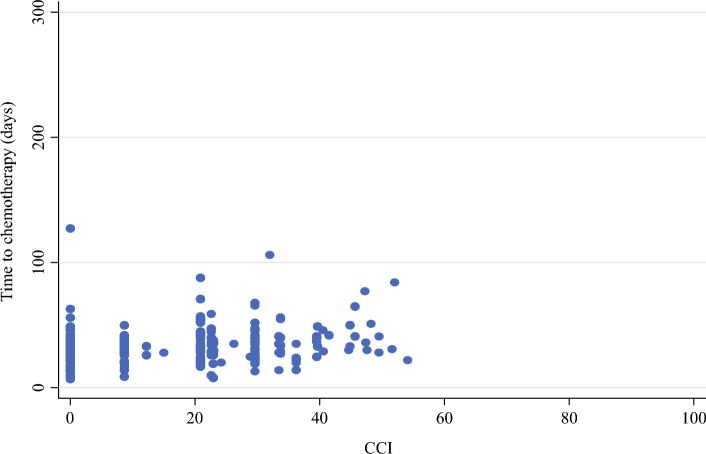
Correction between the complications’ burden and the time to adjuvant chemotherapy start

As shown in Table 3, the median length of hospital stay and the postoperative readmission rates were significantly different among the different complication burden groups. The median hospitalization was 4 days in patients without any postoperative complications (CCI-0), whereas it reached 6, 8.5, and 13 days in the low, intermediate, and high complication burden groups, respectively. Similarly, the postoperative readmission rate was 0% in the CCI-0 group, whereas 44% of patients experienced re-hospitalization in the CCI-high group. Conversely, no statistically significant detrimental effect of complication burden on the median time to the first cycle of adjuvant chemotherapy was observed (*p* = 0.502; Fig. 1).

**Table 3:** Correlation of postoperative complications burden and morbidity indicators

		No complications (CCI-0)		CCI-low		CCI-intermediate		CCI-high		*p*-Value
Length of stay	Median (IQR)	4	4–5	6	4–8	8.5	6.75–11	13	10–18.75	< 0.0001
Postoperative readmission	Yes	0	0%	6	5%	5	16%	18	44%	< 0.0001
	No	104	100%	121	95%	27	84%	23	56%	
Time to chemotherapy	Median (IQR)	31	26–37	33	26–40	35	27.5–44	25	29–44	0.502

Table 4 reports the correlation between surgical complexity and postoperative complication burden evaluated both with the Clavien–Dindo classification and CCI scoring. Although the incidence of postoperative complications (*p* < 0.0001) and the rate of postoperative readmission (*p* = 0.011) significantly differed among the three SCS categories, no statistical difference was found in complication severity among the three SCS groups when using the Clavien–Dindo classification (*p* = 0.051). Conversely, when described using the CCI, complication severity and burden increased with increasing surgical complexity. The median CCI resulted in patients with higher SCS scores compared with both low and intermediate SCS (*p* < 0.0001); similarly, the rate of low, intermediate, and high complication burden significantly differed between the three SCS subgroups (*p* < 0.0001).

**Table 4:** Correlation of postoperative morbidity indicators and surgical complexity

		SCS low (113)		SCS intermediate (121)		SCS high (70)		
Postoperative complications < 30 days	Yes	56	49.6%	85	70.2%	59	84.3%	**< 0.0001**
	No	57	50.4%	36	29.8%	11	15.7%	
Postoperative re-intervention < 30 days	Yes	5	4.4%	10	8.3%	8	11.4%	0.199
	No	108	95.6%	111	91.7%	62	88.6%	
Postoperative hospital readmission	Yes	6	5.3%	9	7.4%	13	18.6%	**0.011**
	No	107	94.7%	112	92.6%	57	81.4%	
Peri/postoperative blood transfusions	Yes	25	22.1%	54	44.6%	42	60.0%	**< 0.0001**
	No	88	77.9%	67	55.4%	38	54.3%	
Patients with at least one postoperative complication < 30 days	Grade I	23	20.4%	30	24.8%	19	27.1%	0.202
	Grade II	30	26.5%	67	55.4%	52	74.3%	
	Grade III	6	5.3%	17	14.0%	17	24.3%	
	Grade IV	0	0.0%	2	1.7%	2	2.9%	
	Grade V	2	0.7%	0	0.0%	1	1.4%	
Clavien–Dindo classification of complications	Grade I	19	33.9%	13	15.3%	8	13.6%	0.051
	Grade II	29	51.8%	55	64.7%	34	57.6%	
	Grade III	6	10.9%	15	17.6%	14	23.7%	
	Grade IV	0	0.0%	2	2.4%	2	3.4%	
	Grade V	2	3.6%	0	0.0%	1	1.7%	
CCI	Median (IQR)	0	0–20.9	20.9	0–29.6	22.6	11.3–36.2	**< 0.0001**
	CCI-low	45	80.4%	52	61.9%	30	50-8%	**< 0.0001**
	CCI-intermediate	4	7.1%	18	21.4%	10	16.9%	
	CCI-high	7	12.5%	15	12.4%	19	32.2%	

Bold values are statistically significant results

### Predictors of Complications

Age, BMI, ASA score, ECOG PS, comorbidities, previous abdominal surgery, preoperative Hb, platelet (PLT) count, serum proteins and albumin, FIGO stage, primary surgical treatment, RD at surgery, SCS, diaphragmatic stripping and/or resection, multiple bowel resections, lymphadenectomy, duration of surgery, and intraoperative blood loss were the selected covariates for the evaluation of predictors of complications in univariate analysis (Table 5).

**Table 5:** Predictive factors of complications (any grade, CCI > 0) and high postoperative complications burden (CCI-high)

		Predictors of CCI > 0						Predictors of CCI-high					
		Univariate analysis			Multivariate analysis			Univariate analysis			Multivariate analysis		
Variable	Level	OR	IC	*p*-Value	OR	IC	*p*-Value	OR	IC	*p*-Value	OR	IC	*p*-Value
Age	> 72	1.1	0.78–1.53	0.596	1.4	0.89–2.19	0.141	0.8	0.50–1.29	0.363	0.86	0.49–1.52	0.608
	57–72												
	< 57 (ref)												
BMI	< 18	0.73	0.31–1.72	0.471				1.06	0.26–4.20	0.939			
	> 30												
	18–30 (ref)												
ASA score	ASA 3–4	1.48	0.89–2.49	0.134	1.41	0.68–2.90	0.356	1.73	0.88–3.37	0.11	1.62	0.74–3.54	0.231
	ASA 1–2 (ref)												
ECOG status	2	1.03	0.66–1.62	0.881				0.761	0.39–1.48	0.418			
	1												
	0 (ref)												
Comorbidities	2 or more	1.42	0.82–2.45	0.214				0.86	0.40–1.83	0.689			
	Less than 2 (ref)												
Previous abdmonial surgery	Yes	1.05	0.65–1.68	0.848				1.63	0.83–3.22	0.159			
	No (ref)												
Pre-op Hb	≥ 10 g/dL	0.35	0.14–0.88	**0.026**	0.12	0.03–0.46	**0.002**	1.76	0.51–6.04	0.367			
	< 10 g/dL (ref)												
Pre-op PLT	> 450	1	0.98–1.02	0.913				0.98	0.92–1.05	0.622			
	< 150												
	150–450 (ref)												
Pre-op serum proteins	≥ 6.4	0.56	0.28–1.10	0.09	1.27	0.44–3.61	0.658	0.52	0.24–1.11	**0.091**	0.7	0.28–1.72	0.432
	< 6.4 (ref)												
Pre-op albumin	≥ 3.5	0.46	0.25–0.84	**0.011**	0.79	0.33–1.87	0.590	0.6	0.29–1.25	0.171			
	< 3.5 (ref)												
FIGO stage	IVA–IVB	1.26	0.82–1.94	0.288				2.39	1.29–4.44	**0.006**	2.57	1.19–5.52	**0.016**
	IIIB–IIIC												
	IIIA (ref)												
Surgical treatment	IDS	0.86	0.52–1.41	0.546				0.49	0.22–1.07	0.073	0.69	0.26–1.82	0.452
	PDS (ref)												
Residual disease	R2	1.19	0.84–1.69	0.332				0.91	0.56–149	0.704			
	R1												
	R0 (ref)												
Surgical complexity score	High	2.31	1.63–3.26	**< 0.0001**	1.7	0.89–3.22	0.106	2.41	1.53–3.82	**< 0.0001**	0.9	0.42–1.93	0.78
	Intermediate												
	Low (ref)												
Diaphragmatic procedure	Yes	1.91	1.10–3.32	**0.022**	0.64	0.26–1.59	0.338	2.16	1.10–4.24	**0.025**	0.89	0.33–2.42	0.823
	No (ref)												
Multiple bowel resections	Yes	4.74	1.39–16.1	**0.013**	1.82	0.44–7.42	0.407	5.31	2.34–12.05	**< 0.0001**	5.61	2.04–15.45	**0.001**
	No (ref)												
Lymphadenectomy	Yes	1.03	0.58–1.84	0.926				1.06	0.48–2.36	0.879			
	No (ref)												
Duration of surgery	> 300	2.69	1.85–3.91	**< 0.0001**	2.36	1.28–4.37	**0.006**	3.37	1.97–5.75	**< 0.0001**	2.08	0.96–4.55	0.065
	180–300												
	< 180 min (ref)												
Blood loss	> 1000	2.71	1.75–4.20	**< 0.0001**	1.98	1.13–3.49	**0.018**	2.38	1.54–3.68	**< 0.0001**	1.93	1.11–3.36	**0.021**
	500–1000												
	< 500 (ref)												

Bold values are statistically significant results

Factors contributing to overall postoperative complications were first evaluated. In univariate analysis, low preoperative Hb count, low preoperative serum albumin levels, higher surgical complexity and duration, the need for diaphragmatic procedures or multiple bowel resections, and higher intraoperative blood loss were significantly associated with the overall incidence of complications. In multivariate analysis, only preoperative Hb levels, the duration of surgery, and intraoperative blood loss remained significantly associated with the occurrence of any grade complications.

Regarding predictors of a high complication burden, univariate analysis showed a significant association between the occurrence of CCI-high complications and higher FIGO stage, greater surgical complexity, diaphragmatic procedures, multiple bowel resections, longer duration of surgery, and increased intraoperative blood loss. The timing of surgical treatment approached—but did not reach—statistical significance. In multivariate analysis, FIGO stage, multiple bowel resections, and blood loss were independent predictors of CCI-high complication burden.

## Discussion

This single-institution retrospective study explored the rate of postoperative complications, the reliability of the CCI as a descriptor of the surgical complications burden, and the predictors of postoperative complications in patients receiving cytoreductive surgery for advanced HGOC.

In literature, the rate of postoperative complications after cytoreductive surgery for advanced OC is extremely variable and ranges from 30 to 75%. In our series, the overall rate of postoperative complications was 65.8%; re-interventions and hospital readmissions occurred in 7.6%, and 9.2% of cases, respectively. Our data are similar to the series by Kuusela et al.^17^ and Angeles et al.^18^, who reported an overall incidence of any grade postoperative complications in 76.2% and 54.3% of patients undergoing cytoreductive surgery for advanced OC, respectively. Conversely, Jiang et al.^14^ and Palmqvist et al.^16^ reported a 29% global rate of postoperative complications, despite similar surgical timing and similar complexity of surgical procedures compared with our series. Some factors must be considered for the interpretation of these results. First, most papers do not clarify which postoperative events were registered as complications. In contrast, in our series, all postoperative events deviating from a normal postoperative course were recorded, and even those events requiring minimal medical intervention (i.e., short courses of antibiotics for urinary tract infections or bed-side medications for wound infection) were included in the analysis. This might explain the discrepancies in the overall incidence of postoperative complications between our series and those reporting significantly lower rates. Second, most of the complications we recorded were mild, and only 44 patients (14.8%) experienced severe complications requiring surgical interventions or ICU admission (CD grade III–IV). These data are consistent with the available literature, where the incidence of severe postoperative complications range from 7 to 26%. Third, two of the most frequent and clinically significant postoperative events occurring after cytoreductive surgery for the treatment of OC are pleural effusion and anastomotic dehiscence. In our series, pleural effusion was the most frequent pulmonary complication and was recorded in 20 patients (6.6%), 9 of which required drainage positioning. Among the 105 patients undergoing at least one bowel resection, an anastomotic leak occurred in 8 cases, with an overall anastomotic dehiscence incidence of 7.6%. Both these data are in line with the two large series of a tertiary gynecologic oncology center reporting a global incidence of pleural effusion drainage and anastomotic leak of 8.6% and 6.8%, respectively.^15,26^ Globally, these considerations show that the discrepancies in the overall incidence of postoperative complications in the literature are mitigated when only severe complications are considered.

In the literature, there is a wide use of a variety of grading systems evaluating postoperative complications that consider only the most severe events, without giving a global representation of the complication burden.^25,27^ We believe that surgical morbidity and postoperative recovery are influenced not only by the occurrence of single life-threatening complications but also by the sum of multiple minor events. In contrast to other complication grading systems, CCI represents the overall patient complication burden.

To our knowledge, this is the largest series exploring the reliability of the CCI in the description of postoperative complications following cytoreductive surgery for advanced HGOC. Using the CCI, we proposed a three-tier grading of complication burden and chose the cutoffs to encompass in the CCI-high group all patients with at least a complication of grade IIIb or greater, or a combination of grade I–IIIa complications leading to a burden as heavy as a reintervention. Most patients were CCI-low, with only 41 (13.5%) patients included in the CCI-high group. Interestingly, our three-tier stratification correlated with both the duration of hospitalization and the readmission rate, which are well-known indicators of postoperative morbidity. In addition, we explored the correlation between surgical complexity and postoperative complications evaluated both with the CD classification and the CCI. We found that the CCI, both as a continuous and discrete variable, better reflected the increase in surgical morbidity associated with more complex surgery compared with the CD classification.

Adequate selection of patients who can benefit from the major surgical procedure of PDS is crucial to enhance oncologic outcomes and reduce morbidity, and eventually indicate candidate patients for NACT and IDS. Therefore, knowledge of the predictors of major postsurgical morbidity is crucial for selecting the most accurate timing of cytoreductive surgery according to each patient’s features. In contrast to other literature series that explored predictive factors of major surgical complications considering CD or Accordion grade > III as an endpoint, we evaluated the predictors of high complication burden with a CCI ≥ 33.7 as our endpoint. In multivariable analysis only the FIGO stage, multiple bowel resections, and intraoperative blood loss were independent predictors of high complication burden.

High-complexity surgical procedures were associated with the occurrence of high complication burden in univariate analysis, but no association was found with RD after cytoreductive surgery. This corroborates our previous findings^13^ demonstrating that the higher rate of complete cytoreduction derived from extensive PDS is not burdened with a significant increase in postoperative morbidity.

Although a trend in favor of IDS was observed, the timing of cytoreductive surgery did not significantly correlate with the occurrence of a CCI-high burden either in univariate or multivariate analysis.

This finding contrasts with the results of previous prospective and retrospective studies, where IDS was associated with a significantly lower rate of severe complications.^7,14,16,18,28^ Although they receive less complex surgical procedures, it might be that patients undergoing IDS are more prone to experience multiple minor events (i.e., postoperative anemia, wound infections, and delayed wound healing) weighing as much as a single intermediate or major complication, and thus affecting the postoperative course. In addition, the NACT-IDS sequence can be chosen as a treatment strategy in the case of impaired physical conditions not permitting PDS. In this scenario, on the one hand, NACT reduces tumor burden, but on the other, hardly impacts patients’ baseline comorbidities and can even be associated with chemotherapy-related morbidity. Therefore, patients receiving IDS harbor the same—if not worse—risk factors that lead clinicians to choose this treatment strategy and might thus be as likely to experience postoperative complications as those receiving PDS. Furthermore, there is a non-negligible percentage of patients who suboptimally respond to NACT. In these patients, disease diffusion might require extensive debulking procedures and surgery might be burdened not only by NACT-related morbidity but also by the extensive surgical procedures required for optimal cytoreduction.

Although it must be further investigated in larger cohorts, and eventually in prospective studies, this finding puts the focus not only on the accuracy of patient selection for proper surgical timing but also on the identification of high-risk patients for adequate perioperative care and adoption of internal evidence-based, protocolled prevention bundles.

Comprehensively, our study shows that the CCI is a good descriptor of postoperative complications in patients receiving cytoreductive surgery for advanced HGOC, and that predictors of high complication burden might differ from those predicting single severe complications. The real-world scenario is one of the major strengths of this work; all consecutive patients treated surgically with cytoreductive intent at our institution in 2015–2023 were included in our analysis to reduce selection bias. We strongly believe that the global complication burden better reflects surgical morbidity rather than considering the most severe complication that occurred, and we considered both the number and the severity of complications in a quantitative score that was extensively validated in non-gynecological cancer surgery. The main limitation of our analysis is its retrospective nature, due to which data collection might have suffered missing or misreported events possibly impacting the results. Furthermore, this is a single-center analysis and its results are hardly generalizable unless larger multicenter, eventually prospective, studies can provide further evidence on the reliability of the CCI and the predictors of high complication burden in patients with advanced HGOC.

## Conclusions

The CCI is a good descriptor of postoperative complications in patients receiving cytoreductive surgery for advanced HGOC and, contrary to other complications grading systems, encompasses both the severity and the number of complications in an easily usable and intuitive quantitative score. Therefore, we strongly encourage its implementation in routine reporting of postoperative complications after HGOC cytoreductive surgery.

Our analysis shows that FIGO stage, multiple bowel resections, and intraoperative blood loss are independent predictors of high complication burden and should be considered for accurate planning, timing, and selection of patients who are candidates for cytoreductive surgery. Conversely, the timing of surgery did not significantly impact patient postoperative morbidity, and accurate identification of NACT-IDS patients at higher risk of complications is desirable to adopt proper prevention bundles.
